# Transoral endoscopic thyroidectomy vestibular approach as a safe and feasible alternative to open thyroidectomy: a systematic review and meta-analysis

**DOI:** 10.1097/JS9.0000000000000444

**Published:** 2023-05-10

**Authors:** Moon Young Oh, Young Jun Chai, Hyeong Won Yu, Su-Jin Kim, June Young Choi, Kyu Eun Lee

**Affiliations:** a Department of Surgery, Seoul National University College of Medicine, Seoul Metropolitan Government - Seoul National University Boramae Medical Center; b Transdisciplinary Department of Medicine and Advanced Technology, Seoul National University Hospital; c Department of Surgery, Seoul National University College of Medicine, Seoul; d Department of Surgery, Seoul National University Bundang Hospital, Seongnam-si, Korea

**Keywords:** open thyroidectomy, transoral endoscopic thyroidectomy vestibular approach, transoral thyroidectomy

## Abstract

**Background:**

Transoral endoscopic thyroidectomy vestibular approach (TOETVA) is a scarless alternative to open thyroidectomy (OT). This systematic review and meta-analysis aimed to synthesize evidence comparing the intraoperative and postoperative outcomes of TOETVA and OT.

**Methods:**

A systematic literature search of PubMed, Web of Science, the Cochrane Library, and Google Scholar was performed to identify studies comparing the outcomes of TOETVA and OT published before February 2023. The outcomes of interest were operative time, intraoperative blood loss, hospital stay, postoperative pain, number of central lymph nodes retrieved, number of metastatic central lymph nodes, and incidences of transient and permanent recurrent laryngeal nerve injury, transient and permanent hypocalcemia, hematoma, and infection.

**Results:**

Thirteen studies published between 2016 and 2022, involving a total of 2889 patients (TOETVA, *n*=1085; OT, *n*=1804) were included in this systematic review and meta-analysis. Meta-analysis showed that the TOETVA group had a significantly longer overall operative time (weighted mean difference [WMD] 55.19; 95% CI, 39.15, 71.23; *P*<0.001), longer hospital stay (WMD, 0.27; 95% CI, 0.14, 0.39; *P*<0.001), and lower pain scores on postoperative day 1 (WMD, −1.41; 95% CI, −2.79, −0.03; *P*=0.04) than the OT group. Other intraoperative and postoperative outcomes were not significantly different between the groups.

**Conclusion:**

TOETVA has a similar safety profile to OT with less postoperative pain, making it an appropriate and more cosmetically appealing alternative to OT for select patients.

## Introduction

HIGHLIGHTS

- Operative time and length of hospital stay were longer in patients who underwent transoral endoscopic thyroidectomy vestibular approach (TOETVA) than those who underwent open thyroidectomy (OT).
- Patients who underwent TOETVA had less postoperative pain on postoperative day 1 than those who underwent OT.
- Intraoperative blood loss, permanent and transient recurrent laryngeal nerve injury, permanent and transient hypocalcemia, number of retrieved central lymph nodes, number of metastatic central lymph nodes, incidence of hematoma, and infection rates were comparable between patients who underwent TOETVA and OT.

Open thyroidectomy (OT) has long been the standard surgical option for patients undergoing thyroidectomy. However, OT leaves a permanent visible scar on the neck, which has led to an increasing demand for surgical options which provide better cosmesis^1^. Various minimally invasive techniques, including endoscopic and robotic approaches, such as the trans-axillary, subclavian, and axillo-breast approaches^2,3^, have been developed and performed to address the shift toward resolving cosmetic concerns^4^. However, some degree of scaring remains, and there are also safety concerns about the wide flap dissection required for some of these methods^5^.

The transoral endoscopic thyroidectomy vestibular approach (TOETVA), first established and described in 2016^6^, is the only ‘truly scarless’ thyroidectomy to date and it requires a smaller area of flap dissection compared to other remote-access surgeries^7^. Several studies report that TOETVA is becoming popular among surgeons owing to its good safety profile, relatively short learning curve, and acceptable inclusion criteria; and gaining favor among patients because of its lower postoperative pain and superior cosmetic results with associated quality of life^8–11^. One meta-analysis, which included six studies conducted in China or Thailand compared the outcomes of TOETVA and OT^12^. Except for the longer operative time and larger volume of drainage in the TOETVA group, the study reported no significant differences between the two approaches across a relatively narrow range of postoperative outcomes. Another recent meta-analysis compared the outcomes of transoral endoscopic/robotic thyroidectomy and OT, reporting that the transoral approaches had comparable outcomes to OT^13^. In general; however, endoscopic and robotic techniques show different operative and safety outcomes^14–16^. Thus, comparing only TOETVA and OT may provide a more valuable comparison of outcomes associated with these specific techniques.

The present systematic review and meta-analysis aimed to synthesize the current evidence comparing a comprehensive range of intraoperative and postoperative outcomes between TOETVA and OT, to provide an updated and broader reflection of studies with larger cohorts conducted worldwide.

## Methods

This study was registered at the Research Registry (Research Registry Registration Number: reviewregistry1563). The systematic review was conducted in accordance with the Preferred Reporting Items for Systematic Reviews and Meta-Analyses (PRISMA) guidelines (Supplementary Material 1, Supplemental Digital Content 1, http://links.lww.com/JS9/A477, Supplemental Digital Content 2, http://links.lww.com/JS9/A478) and the Assessing the Methodological Quality of Systematic Reviews (AMSTAR) guidelines (Supplementary Material 2, Supplemental Digital Content 3, http://links.lww.com/JS9/A479)^17^. Internal review board approval was not required because only previously published data were included in the meta-analysis.

### Systematic literature search

A systematic literature search of studies comparing TOETVA and OT published before February 2023 was performed using the PubMed, Web of Science, the Cochrane Library, and Google Scholar databases with the following medical subject headings and keywords: ‘endoscopy’, ‘transoral endoscopic’, ‘vestibular’, ‘open’, ‘conventional open’, and ‘thyroidectomy’, or ‘thyroid surgery’. After removing duplicates, two independent reviewers (M.Y.O. and Y.J.C.) screened the titles and abstracts of the initial search findings to identify candidate studies for inclusion. Candidate studies were retrieved and assessed for eligibility based on the inclusion and exclusion criteria. The final decision for inclusion was determined based on consensus between the two researchers.

### Inclusion and exclusion criteria

The inclusion criteria were as follows: studies that were human clinical studies, were published in English with full-text descriptions, clearly compared TOETVA and OT, and reported at least one intraoperative or postoperative outcome of interest. The exclusion criteria were publications that were abstracts, reviews, case reports, letters, editorials, or expert opinions, studies that were noncomparison studies, comparisons of transoral robotic thyroidectomy and OT, and those that did not report the outcomes of interest.

### Outcome measures of interest

The intraoperative outcomes of interest were operative time, intraoperative blood loss volume, number of retrieved central lymph nodes (CLN), and number of metastatic lymph nodes. The postoperative outcomes of interest were incidences of transient and permanent recurrent laryngeal nerve (RLN) injuries, transient and permanent hypocalcemia, hematoma, and infection, as well as length of hospital stay and postoperative day (POD) 1 pain score using the visual analog scale (VAS).

### Data extraction and quality assessment

Two independent reviewers (M.Y.O. and Y.J.C.) extracted the data using a standardized form, which included patient characteristics and intraoperative and postoperative outcomes. The Newcastle–Ottawa Scale (NOS) was used to assess each study’s quality of selection of treatment groups, comparability of treatment groups, and exposure of outcomes^18^. Studies with a NOS score of five or more stars were considered to have adequate quality for analysis.

### Certainty of evidence

Grading of Recommendations Assessment, Development, and Evaluation (GRADE) was performed by two independent reviewers (M.Y.O. and Y.J.C.) to assess the certainty of evidence, using GRADE Pro Software (McMaster University and Evidence Prime Inc)^19^.

### Statistical analysis

Meta-analysis was conducted using Review Manager software version 5.4.1 (The Cochrane Collaboration). A weighted mean difference (WMD) with a corresponding 95% CI was used to express continuous outcomes, and odds ratio (OR) with corresponding 95% CI was used to express categorical outcomes. When non-normally distributed continuous data was observed, a standardized mean difference with a corresponding 95% CI was used to express continuous outcomes. For continuous data not in the form of mean or standard deviation, the estimated mean and standard deviation from the sample size, median, range, and/or interquartile range were calculated using methods described by Wan *et al* ^20^. Heterogeneity was determined by the *I* ^2^ value, with a *P*-value<0.05 interpreted as significant. Fixed or random effects models were used to calculate the pooled outcome measures, depending on the degree of heterogeneity. A fixed effects model was used if the *I* ^2^ value was less than 50%, and a random effects model was used if the *I* ^2^ value was more than 50%. Sensitivity analyses were performed by excluding each individual study for each outcome measure analysis to observe the relative effect on the overall results, following the same criteria for fixed and random effects models. Funnel plots were plotted for outcomes with 12 or more studies included to evaluate potential publication bias.

## Results

### Study characteristics

**Figure 1:**
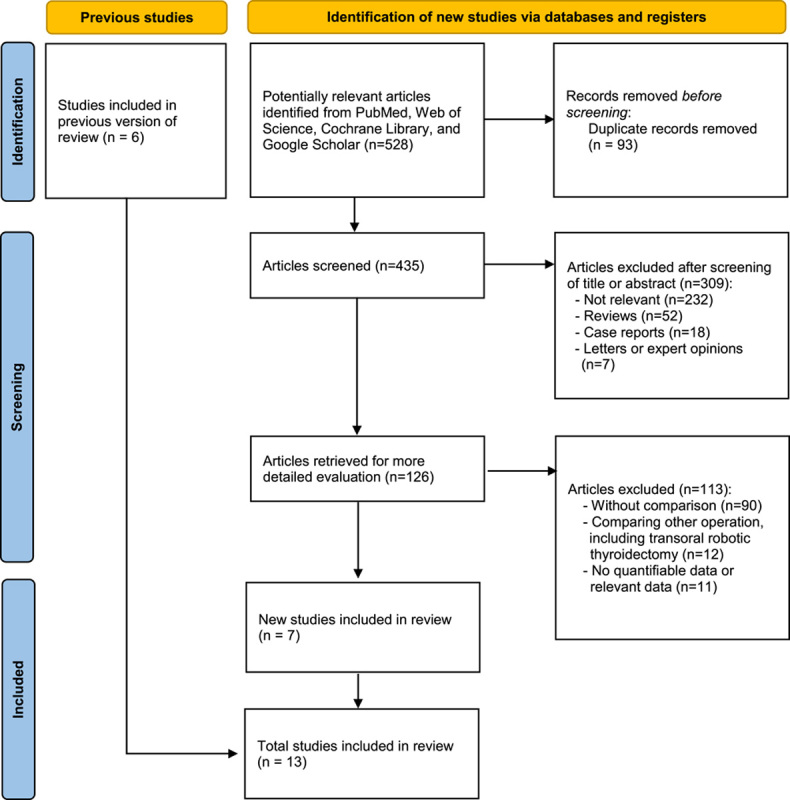
Flow diagram shows the process of study selection.

The initial systematic literature search yielded 528 articles. Ninety-three duplications were removed, leaving 435 articles for title and abstract screening. A further 309 studies were excluded, leaving 126 articles for detailed evaluation of the full-text. Finally, 13 studies were included in the systematic review and meta-analysis (Fig. 1). All were nonrandomized studies that were published between 2016 and 2022. The articles included a total of 2889 patients: 1085 patients who underwent TOETVA and 1804 patients who underwent OT. Table 1 describes the study characteristics and patient characteristics by surgical approach.

**Table 1:** Study characteristics and patient characteristics by surgical approach.

References	Study period	Country	Design	Groups	Number of patients	Age (years)	Sex (F/M)	Pathology (Malignant/Benign)	Nodule size (mm)	Type of surgery (TT/Lobectomy)
Jipratoom^21^	2014–2016	Thailand	Retrospective	TOETVA	45	32.84±9.01	41/5	0/45	N/A	N/A
				OT	49	32.79±9.53	44/5	0/49	N/A	N/A
Anuwong^22^	2014–2016	Thailand	Retrospective	TOETVA	216	35.1±11.9	199/20	N/A	4.10±1.60	86/133
				OT	216	35.3±12.1	197/19	N/A	4.70±1.90	84/132
Bian^23^	2014–2016	China	Retrospective	TOETVA	30	24.0 (15.5, 32.5)	93/6	30/0	1.50 (1.00, 2.00)	N/A
				OT	30	26.5 (20.2, 32.8)	43/12	30/0	1.25 (0.52, 1.98)	N/A
Perez-Soto^24^	2017–2019	Mexico	Retrospective	TOETVA	20	48.10±15.67	18/2	18/2	1.71 (0.46, 2.96)	16/4
				OT	20	45.55±14.42	N/A	18/2	1.67 (0.50, 2.84)	17/3
Ahn^25^	2016–2019	Korea	Retrospective	TOETVA	150	43.06±10.90	145/5	138/12	0.91 (0.24, 1.59)	40/110
				OT	125	51.02±12.42	89/36	123/2	1.19 (0.47, 1.91)	85/40
Han^26^	2017–2019	Korea	Retrospective	TOETVA	52	39.7±10.0	50/2	46/6	0.90±0.6	N/A
				OT	50	45.1±10.5	39/11	43/7	1.10±1.0	N/A
Kasemsiri^27^	2017–2018	Thailand	Retrospective	TOETVA	32	38.3±11.3	32/0	1/31	2.80±1.20	N/A
				OT	38	46.7±10.9	34/4	0/38	3.40±1.20	N/A
Sun^28^	2017–2018	China	Retrospective	TOETVA	100	29.65±6.57	86/14	100/0	0.71±0.38	N/A
				OT	289	45.18±11.47	165/124	289/0	0.70±0.35	N/A
Wang^29^	2015–2016	China	Retrospective	TOETVA	80	31.48±6.60	80/0	80/0	0.87±0.56	N/A
				OT	80	32.59±5.8	80/0	80/0	0.76±0.47	N/A
Liu^30^	2017–2020	China	Retrospective	TOETVA	96	28 (25, 32)	80/16	142/0	1.40 (1.20, 1.50)	21/75
				OT	425	42 (34, 49)	267/158	43/0	1.30 (1.13, 1.60)	213/212
Russell^33^	2017–2020	USA	Retrospective	TOETVA	200	39 (16, 71)^*^	177/23	65/135	3.0 (0.4, 7.1)^*^	72/128
				OT	333	47 (10, 84)^*^	266/67	136/196	2.3 (0.4, 6.0)^*^	168/165
Nguyen^32^	2020–2021	Vietnam	Retrospective	TOETVA	47	38.0±10.5	46/1	0/47	2.57±1.05	4/43
				OT	31	52.5±13.4	3/28	0/31	2.65±12.5	8/23
Van Den Heede^31^	2020–2021	France	Retrospective	TOETVA	34	44.9±12.5	100/0	6/28	21 (5, 25)	0/21(+13 isthmectomy)
				OT	340	44.9±12.6	259/81	127/213	12 (7, 30)	0/332(+8 isthmectomy)

Data are presented as mean±SD or median (with interquartile range) or.

* Median (with minimum and maximum values), unless stated otherwise.

N/A, Not available; OT, Open thyroidectomy; TOETVA, Transoral endoscopic thyroidectomy vestibular approach; TT, total thyroidectomy.

### Intraoperative outcomes

#### Operative time

**Figure 2:**
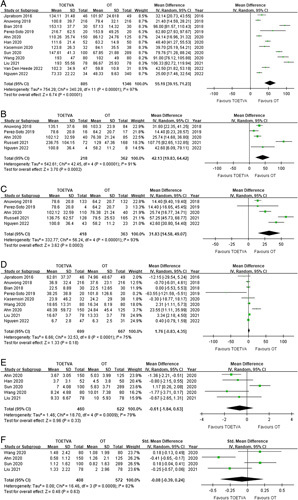
Forest plots comparing intraoperative outcomes between TOETVA and TORT. 2A, Forest plot shows comparison of the overall operative time in minutes. 2B, Forest plot shows comparison of lobectomy time in minutes. 2C, Forest plot shows comparison of total thyroidectomy time in minutes. 2D, Forest plot shows comparison of the intraoperative blood loss in ml. 2E, Forest plot shows comparison of the number of retrieved central lymph nodes. 2F, Forest plot shows comparison of the number of retrieved central lymph nodes.

Twelve studies provided reported operative time for TOETVA and OT groups^21–32^. Pooled analysis showed that the TOETVA group had a significantly longer overall operative time than the OT group, with a WMD of 55.19 min (95% CI, 39.15, 71.23; *P*<0.001). Significant heterogeneity was noted (*I* ^2^=97%, *P*<0.001) (Fig. 2A). Five studies performed separate analyses of operative time according to the extent of surgery^22,24,25,32,33^. The TOETVA group had a significantly longer operative time for both total thyroidectomy (WMD, 42.13; 95% CI, 19.83, 64.42; *P*<0.001) and lobectomy alone (WMD, 31.83; 95% CI, 14.58, 49.07; *P*<0.001) (Fig. 2B, C).

#### Intraoperative blood loss

Nine studies reported intraoperative blood loss for TOETVA and OT groups^21–25,27,29,30,32^. Pooled analysis of the data revealed that the TOETVA and OT groups did not differ significantly in terms of intraoperative blood loss (WMD 1.76; 95% CI -0.83, 4.35; *P*=0.18), and significant heterogeneity was noted (*I* ^2^=75%, *P*<0.001) (Fig. 2D). Anuwong *et al.* ^22^ and Nguyen *et al.* ^32^ also provided information about intraoperative blood loss according to the extent of surgery and both showed that the volume of blood loss did not significantly differ between TOETVA and OT groups for total thyroidectomy or lobectomy alone.

#### Central lymph nodes

Five studies reported the number of CLNs retrieved during the surgical procedure for TOETVA and OT groups^25,26,28–30^. Pooled analysis of the data showed that the number of CLNs retrieved was not significantly different between the TOETVA and OT groups (WMD, −0.61; 95% CI, −1.84, 0.63; *P*=0.32), and significant heterogeneity was observed (*I* ^2^=79%, *P*<0.001) (Fig. 2E). The number of metastatic CLNs for TOETVA and OT groups was reported by four studies and there was no significant difference between groups (standardized mean difference, –0.08; 95% CI, –0.39, 0.24; *P*=0.63), and significant heterogeneity was noted (*I* ^2^=82%, *P*<0.001) (Fig. 2F)^25,28–30^.

### Postoperative outcomes

#### Recurrent laryngeal nerve injury

**Figure 3:**
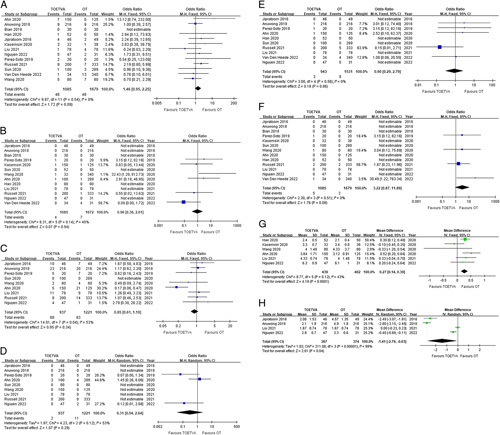
Forest plots comparing postoperative outcomes between TOETVA and TORT. 3A, Forest plot shows comparison of transient recurrent laryngeal nerve injuries. 3B, Forest plot shows comparison of permanent recurrent laryngeal nerve injuries. 3C, Forest plot shows comparison of transient hypocalcemia. 3D, Forest plot shows comparison of permanent hypocalcemia. 3E, Forest plot shows comparison of hematomas. 3F, Forest plot shows comparison of infections. 3G, Forest plot shows comparison of hospital stay in days. 3H, Forest plot shows comparison of postoperative day 1 VAS pain score.

All 13 studies reported the incidences of transient and permanent RLN injury for the TOETVA and OT groups^21–33^. Pooled analysis showed no significant differences between TOETVA and OT groups for both transient RLN injury (OR, 1.46; 95% CI, 0.95, 2.25; *P*=0.09), without significant heterogeneity (*I* ^2^=0%, *P*=0.54) (Fig. 3A); and permanent RLN injury (OR, 0.96; 95% CI, 0.36, 2.61; *P*=0.94), with significant heterogeneity (*I* ^2^=40%, *P*=0.14) (Fig. 3B). Han *et al.* ^26^ conducted a study comparing subjective and objective postoperative voice outcomes by using questionnaires and acoustic and aerodynamic analyses, and the results showed that there were no significant changes in subjective or objective voice outcomes between TOETVA and OT groups at 3 and 6 months after surgery.

#### Hypocalcemia

Nine studies reported the incidences of transient and permanent hypocalcemia for TOETVA and OT groups^21,22,24–26,30–33^. Eight studies were included in the pooled analysis of transient hypocalcemia^21,22,24,25,29,30,32,33^, and three studies were included for permanent hypocalcemia^24,25,32^ (the remaining studies reported no incidences of hypocalcemia in either arm). There were no statistically significant differences between the TOETVA and OT groups for transient or permanent hypocalcemia (OR 0.85; 95% CI 0.61, 1.19; *P*=0.34 and OR 0.31; 95% CI 0.04, 2.64; *P*=0.29, respectively). Significant heterogeneity was seen for transient hypocalcemia (*I* ^2^=53%, *P*=0.04) but not for permanent hypocalcemia (*I* ^2^=53%, *P*=0.12) (Fig. 3C, D).

#### Hematoma

Ten studies reported the incidences of hematoma for TOETVA and OT groups^21,22,24–26,28,30–33^, five of which reported zero events for both TOETVA and OT groups^21,26,28,30,32^. Pooled analysis of the remaining five studies showed no statistically significant differences between groups (OR 0.90; 95% CI 0.29, 2.79; *P*=0.86), and no significant heterogeneity was noted (*I* ^2^=0%, *P*=0.56) (Fig. 3E)^22,24,25,31,33^.

#### Perioperative antibiotics administration and infection

Eight studies reported the use of perioperative antibiotics for TOETVA and OT groups^21,22,24,27–31^. All eight studies reported that only the TOETVA group received antibiotics while the OT group did not. Two studies reported the administration of prophylactic preoperative intravenous antibiotics only^27,29^, while six studies reported the use of preoperative intravenous antibiotics which were continued for between 24 h and 9 days postoperatively^21,22,24,28,30,31^. Four studies provided specific information about the type of antibiotics used: three reported administration of amoxicillin-clavulanic acid^21,22,31^, and one study reported administration of ceftriaxone or cephalotin with metronidazole^24^.

All 13 studies reported the incidences of infection for the TOETVA and OT groups^21–33^. Nine studies reported zero events for both groups^21–23,25–28,30,32^. Pooled analysis of the remaining four studies showed a higher incidence of infection in the TOETVA group than the OT group, but without a statistically significant difference (OR, 3.22; 95% CI, 0.87, 11.89; *P*=0.08)^24,29,31,33^. No significant heterogeneity was noted (*I* ^2^=0%, *P*<0.51) (Fig. 3F). Of the two studies that reported the use of preoperative antibiotics alone^27,29^, one study reported one infection event (1/80, 1.25%) in the TOETVA group^29^. Among the six studies that reported use of both preoperative and postoperative antibiotics in the TOETVA group only^21,22,24,28,30,31^, one study reported one infection event (1/20, 5.00%) in the TOETVA group^24^ and one other study reported one infection even (1/34, 2.94%) in the OT group^31^. A study with no information about the use of antibiotics reported two infection events each in the TOETVA group (2/200, 1%) and the OT group (2/333, 0.6%)^33^. No infection events were reported in the other studies, including those that did not provide information about the use of antibiotics^21–23,25–28,30,32^.

#### Hospital stay

Six studies reported the duration of hospital stay for TOETVA and OT groups^25–27,29,30,32^. Pooled analysis showed that the TOETVA group had a longer hospital stay than the OT group (WMD, 0.27; 95% CI, 0.14, 0.39; *P*<0.001), and no significant heterogeneity was observed (*I* ^2^=43%, *P*=0.12) (Fig. 3G).

#### Postoperative pain

Four studies reported POD 1 VAS pain scores for TOETVA and OT groups^21,22,30,32^. Pooled analysis of the data revealed that the TOETVA group experienced less pain than the OT group (WMD, –1.41; 95% CI, −2.79, −0.03; *P*=0.04) ), and significant heterogeneity was noted (*I* ^2^=99%, *P*<0 001) (Fig. 3H). Additionally, two studies showed that VAS pain scores for POD 1, 2, 3, and the overall average of all three days in the TOETVA group were significantly less than those of the OT group^21,22^. One study assessed postoperative VAS pain scores after POD 3 and showed that POD 4 pain scores were significantly lower in the TOETVA group compared to the OT group, and that there were no significant differences in pain scores between the two groups on POD 7^32^. Although not included in the POD 1 VAS pain score analysis, the study by Kesemsiri *et al.* ^27^ showed that the VAS pain scores for postoperative 2, 6, and 12 weeks were not significantly different between the TOETVA and OT groups.

### Sensitivity and subgroup analyses

Sensitivity analyses were carried out by excluding each individual study from the data set for each of the outcome measures. The primary sources of heterogeneity for permanent RLN injury, transient and permanent hypocalcemia, and the number of CLNs retrieved were identified. The *I* ^2^ value for permanent RLN injury was reduced from 53 to 0% after excluding either the study by Wang *et al.* ^29^ or the study by Van Den Heede *et al.* ^31^. The result for transient RLN injury changed after excluding the study by Wang *et al.* ^29^ (WMD, 1.64; 95% CI, 1.03, 2.62; *P*=0.04) and also changed after excluding the study by Liu *et al.* ^30^ (WMD, 1.62; 95% CI, 1.04, 2.53; *P*=0.03). The *I* ^2^ value for both transient hypocalcemia and permanent hypocalcemia was reduced from 53 to 0% after excluding one study^25^, without changing the conclusion. The result also changed for the number of CLNs retrieved after excluding one study^28^ (WMD, −1.21; 95% CI, −1.85, −0.57; *P*<0.001). Influence analysis of the other outcomes did not show any significant changes in the results.

Subgroup analyses were performed on studies which only included malignancy^23,28–30^, and those that only included benign lesions or disease^21,27,32^. The *I* ^2^ value for intraoperative blood loss was reduced from 75 to 0% for both subgroup analyses of studies that only included malignancy and studies that only included benign lesions or disease. The meta-analysis of subgroups showed results consistent with the meta-analysis of the overall pooled analysis (Table 2).

**Table 2:** Meta-analysis of subgroups.

Outcomes	Number of studies	Number of patients	OR/WMD	95% CI	*P*	*I* ^2^ (%)
Studies only including malignancy	4					
Operative time	3	765	92.57	80.14, 105.01	<0.001	74
Blood loss	3	376	3.11	2.03, 4.19	<0.001	0
Retrieved CLN	3	705	−0.27	−2.17, 1.64	0.78	77
Metastatic CLN	3	705	0.05^*^	−0.22, 0.32	0.73	64
Transient RLN palsy	3	705	0.58	0.23, 1.48	0.26	0
Transient hypocalcemia	2	316	1.00	0.44, 2.25	1.00	0
Hospital stay	2	316	0.23	−0.11, 0.57	0.18	46
Studies only including benign lesions or disease	3					
Operative time	3	539	30.51	22.34, 38.67	<0.001	42
Blood loss	3	243	0.34	−0.85, 1.52	0.57	0
Transient RLN palsy	3	243	2.28	0.78, 6.70	0.13	0
Transient hypocalcemia	2	173	1.86	0.72, 4.83	0.20	0
Hospital stay	2	148	0.02	−0.26, 0.29	0.90	9
Postoperative pain (POD 1)	2	173	−1.43	−3.48, 0.62	0.17	97

* Standardized mean difference.

CLN, central lymph nodes; OR, odds ratio; POD, postoperative day; WMD, weighted mean difference.

### Quality assessment and publication bias

**Figure 4:**
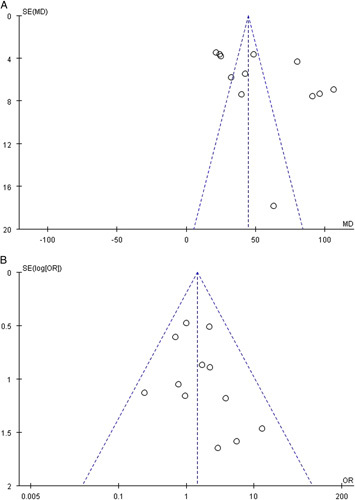
Funnel plot to investigate publication bias. 4A, Funnel plot of 12 studies based on operative time. 4B, Funnel plot of 12 studies based on transient recurrent laryngeal nerve injuries.

NOS was used to assess the comparability of each included study, and yielded scores which ranged from five to eight (Table 3). According to the GRADE assessment of seven main outcomes, the certainty of evidence ranged from very low to moderate (Supplementary Material 3, Supplemental Digital Content 4, http://links.lww.com/JS9/A480 and 4, Supplemental Digital Content 5, http://links.lww.com/JS9/A481). Funnel plots of the operative time and transient RLN injuries outcomes are shown in Figure 3. The operative time funnel plot included 12 studies and showed an asymmetrical distribution, suggesting probable publication bias (Fig. 4A). Twelve studies were included for the transient RLN injuries funnel plot, because one study had zero incidences in both arms. Although none of the twelve studies lay outside the limits of the 95% CI of the funnel plot, there was a somewhat asymmetric distribution of the studies around the vertical axis, suggesting moderate publication bias (Fig. 4B).

**Table 3:** Newcastle–ottawa scoring system for nonrandomized comparative studies.

References	Selection star	Comparability star	Outcome Star	Total star
Jipratoom^21^	3	1	2	6
Anuwong^22^	3	2	1	6
Bian^23^	2	2	1	5
Perez-Soto^24^	3	1	1	5
Ahn^25^	3	2	2	7
Han^26^	3	2	2	7
Kasemsiri^27^	3	2	2	7
Sun^28^	3	2	3	8
Wang^29^	2	2	2	6
Liu^30^	3	2	3	8
Russell^33^	3	1	1	5
Nguyen^32^	3	2	2	7
Van Den Heede^31^	3	2	2	7

## Discussion

TOETVA is a remote-access approach for thyroidectomy, which produces scar-free results and favorable postoperative outcomes^34^. A previous meta-analysis that compared TOETVA with standard OT reported that TOETVA is a feasible and safe alternative procedure to OT^12^. Owing to the steadily rising popularity of TOETVA, several subsequent studies conducted in different countries have been published. A recently published meta-analysis compared outcomes between transoral thyroidectomy and OT, but included both TOETVA and robotic approaches^13^. We conducted a dedicated study specifically comparing TOETVA and OT in order to update the findings as well as explore further outcomes, such as the incidence of postoperative hematoma and POD 1 pain scores.

TOETVA was associated with a longer overall operative time, as well as a longer time for total thyroidectomy and lobectomy compared with OT. The longer operative time required by TOETVA may be due to the additional time required to set up the laparoscopic instruments and to create the working space^29^. Although TOETVA is becoming more frequent, it is still a relatively new technique which requires experience and training for the surgeon to overcome the learning curve^35^. This is especially true for those who are unfamiliar with endoscopic surgeries or the top-down view of the transoral approach. We anticipate that the operative time of TOETVA may decrease over time as the operative proficiency and cumulative experience of surgeons and surgical teams increases^16^. Improvements to the TOETVA technique, such as using balloon dilators for easy and rapid flap dissection during TOETVA, may also lead to a reduction in the time needed to create working space, resulting in a shorter overall operative time^36^.

The top-down surgical view afforded by TOETVA may initially be challenging for the surgeon. However, once the surgeon becomes familiar with the technique, cranial to caudal dissection of the RLN may be easier using TOETVA compared to other approaches with different views, because the RLN is approached first at its insertion site then followed down its pathway with good visualization^37^. A previous study also demonstrated that the top-down perspective makes the removal of CLNs easier^28^. Our meta-analysis showed no significant differences in the incidences of transient or permanent RLN injuries or the number of CLNs retrieved between the TOETVA and OT groups. However, the results may have been affected by publication bias based on the possibility that studies were published with the intention of showing comparable results between TOETVA and OT, and that results or studies that showed higher complications in either group may have been withheld.

Based on our review, until POD 4, patients who underwent TOETVA reported less postoperative pain than those who underwent OT. The pain scores became comparable after POD 7. Less pain during the early postoperative period in TOETVA may be because oral vestibule incisions cause less pain than skin incisions on the neck. Compared to the skin, oral wounds contain fewer immune mediators and profibrotic mediators, and more bone marrow-derived cells, as well as a higher re-epithelialization rate, and faster proliferation of fibroblasts than dermal wounds, leading to faster healing of the oral mucosa after injury without scar tissue^38^. Having a more comprehensive insight into the nature of postoperative pain following the two approaches, which investigates postoperative pain scores at various locations, such as the anterior neck, lateral neck, lower lip, and chin, as well as swallowing pain, would be helpful.

Despite having lower postoperative pain scores, patients who underwent TOETVA had longer hospital stays than those who underwent OT, presumably because of a longer recovery time. This may be owing to the longer operative time resulting from the larger area of flap dissection required by TOETVA. On the other hand, the length of hospital stay may also be dependent on the culture, health care systems, and cost of surgery in the country or location where the surgery was performed^22^. Additionally, because TOETVA is a relatively new technique, surgeons may have routine policies requiring longer hospital stays to enable extended observation, or patients may request a longer hospitalization.

No direct comparisons of mental nerve injuries were made between TOETVA and OT because mental nerve injury is a transoral approach-specific complication^30^, (the path of dissection in OT does not involve the course of the mental nerve)^27^. However, the incidences of mental nerve injury for TOETVA alone were reported by eight studies, four of which reported zero incidences of any kind of mental nerve injury^21,25,30,31^. The remaining four studies reported transient mental nerve injury incidences in 1.4 to 56.7% of patients^22–24,32^. All patients who had transient mental nerve injury showed full recovery and thus, there were no cases of permanent mental nerve injury in any of the studies. It is important to explore ways for mitigating such injuries. Studies suggest that the lateral incisions should be made in the mucosa at the level of the first premolars and closer to the lower lip to reduce mental nerve injury^30,37^.

While OT has already been recognized as a clean surgery which does not require prophylactic antibiotics, many surgeons choose to use prophylactic antibiotics prior to performing TOETVA because of the perceived risk of infection from the normal flora in the mouth^39^. Of the eight studies that reported the use of antibiotics, all patients who underwent TOETVA received either preoperative or perioperative antibiotics^21,22,24,27–31^. Among the eight studies, only two infection events (0.3%) were reported among 606 patients who underwent TOETVA^24,29^, and one infection event (0.04%) was reported among 1110 patients who underwent OT^31^. These findings suggest that TOETVA is comparable to OT when antibiotics are used in TOETVA. Nevertheless, because there is insufficient evidence to determine the effect of antibiotics in TOETVA, further studies are needed to evaluate the use of antibiotics in TOETVA.

While other remote-access approaches leave some form of scar on the body, TOETVA leaves no visible scars. TOETVA has several other advantages. Other remote-access approaches require extensive flaps, which may increase the risk of bleeding, subcutaneous emphysema, and pneumothorax^40^. Although TOETVA is not invulnerable to these complications, among the remote-access approaches, it provides the shortest distance from the incision to the target site, and thus requires less dissection of flaps to form the working space, conceivably reducing the risk^37^. Furthermore, TOETVA provides a symmetrical midline view of the surgical field, which facilitates easy bilateral nerve visualization and simplifies total thyroidectomy compared to some other remote-access approaches^41^. A systematic review and meta-analysis comparing TOETVA and other remote-access approaches showed that TOETVA are similar in terms of postoperative outcomes such as blood loss, RLN injury, hypocalcemia, duration of hospital stay, seroma, and wound infection^42^.

Cosmetic results and related quality of life are important because these are the primary reasons that TOETVA was developed. One study reported significantly higher cosmetic satisfaction scores among patients who underwent TOETVA than those who underwent OT (3 vs. 2, *P*<0.001)^30^. Similarly, Kesemsiri *et al.* ^27^ reported that two weeks after surgery, TOETVA patients reported better outcomes in quality of life parameters such as physical activity reduction, psychosocial impairment, role physic, and emotion. Nguyen *et al.* ^32^ also reported that 80.9% of patients who underwent TOETVA were ‘very satisfied’ three months after surgery compared to 35.4% of those who underwent OT (*P*<0.001), although the elements of satisfaction were unspecified. Assessments of cosmetic satisfaction and quality of life after surgery were not covered in our meta-analysis. Only three studies provided information about cosmetic results, patient satisfaction, and quality of life, and various evaluation tools and questionnaires were used, making the pooling of data unfeasible^27,30,32^. Future studies using a standardized questionnaire or tool to clearly measure such outcomes are needed to perform a meta-analysis.

This study has several other limitations. All included studies were nonrandomized retrospective studies. TOETVA and OT approaches are difficult to randomize in patients because TOETVA is generally more costly than OT, and patients tend to choose their preferred surgical method based on their personal preferences^43^. Prospective, randomized controlled studies are needed to eliminate many biases that may occur in nonrandomized studies. One limitation of this meta-analysis is the heterogeneity observed in some of the outcomes, which may be attributed in part to the various diseases for which thyroidectomy procedures were performed. Differences in the extent of surgery and operative time may also vary depending on the underlying condition of the patient. Although the disparities of underlying disease in the studies may have contributed to the observed heterogeneity in some of the outcomes, it also highlights the versatility of TOETVA for the treatment of various diseases.

## Conclusion

Patients who underwent TOETVA had longer operative times and hospital stays, and lower postoperative pain scores compared to those who underwent OT. There were no significant differences in other intraoperative or postoperative outcomes, including intraoperative blood loss, transient or permanent RLN injury, transient or permanent hypocalcemia, number of retrieved CLNs or metastatic CLNs, or incidences of hematoma or infection. Based on our results, TOETVA is a safe and feasible alternative approach to OT for select patients.

## Ethical approval

This study was registered at the Research Registry (Research Registry Registration Number: reviewregistry1563): https://www.researchregistry.com/browse-theregistry#registryofsystematicreviewsmetaanalyses/registryofsystematicreviewsmetaanalysesdetails/640e95d8af03920028c85017/. Internal review board approval was not required because only previously published data were included in the meta-analysis.

## Sources of funding

All of the authors (M.Y.O., Y.J.C., H.W.Y., S.J.K., J.Y.C., K.E.L.) state that they have received no funding for this research.

## Author contribution

M.Y.O. and Y.J.C. contributed to the study design, data collection. M.Y.O analyzed the data and wrote the manuscript in consultation with Y.J.C. H.W.Y., S.J.K., Y.J.C., K.E.L. provided critical feedback and helped shape the research, analysis, and manuscript.

## Conflicts of interest disclosure

None.

## Guarantor

The Guarantor for this article is the corresponding author, Young Jung Chai.

## Data availability statement

The data that support the findings of this study are available within the article and its supplementary materials, as well as in the cited references included in the systematic review and meta-analysis.

## Provenance and peer review

Not commissioned, externally peer-reviewed.

## Presentation

None.

## Financial support and sponsorship

None.

## Acknowledgements

None.

## Supplementary Material
