# Supplementary material for: Transoral endoscopic thyroidectomy vestibular approach as a safe and feasible alternative to open thyroidectomy: a systematic review and meta-analysis
Source: Int J Surg. 2023 May 10;109(8):2467–77. doi: 10.1097/JS9.0000000000000444 (PMC10442077; doi:10.1097/JS9.0000000000000444)
Supplement: Supplementary file 2 [file js9-109-2467-s002.docx]

**Previous studies**

**Identification of new studies via databases and registers**

Studies included in previous version of review (n = 6)

Potentially relevant articles identified from PubMed, Web of Science, Cochrane Library, and Google Scholar (n=528)

Records removed *before screening*:

Duplicate records removed (n = 93)

**Identification**

Articles excluded after screening of title or abstract (n=309):

- Not relevant (n=232)

- Reviews (n=52)

- Case reports (n=18)

- Letters or expert opinions

(n=7)

Total studies included in review

(n = 13)

Articles retrieved for more detailed evaluation (n=126)

Articles screened (n=435)

**Screening**

Articles excluded (n=113):

- Without comparison (n=90)

- Comparing other operation,

including transoral robotic

thyroidectomy (n=12)

- No quantifiable data or

relevant data (n=11)

New studies included in review

(n = 7)

**Included**

*From:*  Page MJ, McKenzie JE, Bossuyt PM, Boutron I, Hoffmann TC, Mulrow CD, et al. The PRISMA 2020 statement: an updated guideline for reporting systematic reviews. BMJ 2021;372:n71. doi: 10.1136/bmj.n71

For more information, visit: <http://www.prisma-statement.org/>
