# Supplementary material for: Transoral endoscopic thyroidectomy vestibular approach as a safe and feasible alternative to open thyroidectomy: a systematic review and meta-analysis
Source: Int J Surg. 2023 May 10;109(8):2467–77. doi: 10.1097/JS9.0000000000000444 (PMC10442077; doi:10.1097/JS9.0000000000000444)
Supplement: Supplementary file 4 [file js9-109-2467-s004.pdf]

Author(s): Moon Young Oh, Young Jun Chai
Question: TOETVA compared to OT for thyroidectomy
Setting:
Bibliography:

Certainty assessment							№ of patients		Effect		Certainty	Importance
№ of studies	Study design	Risk of bias	Inconsistency	Indirectness	Imprecision	Other considerations	TOETVA	OT	Relative (95% CI)	Absolute (95% CI)		
**Operation time**												
12	observational studies	not serious	not serious	not serious	not serious	none	885	1346	-	MD **55.19 higher** (39.15 higher to 71.23 higher)	⊕⊕◯◯ Low	
**Transient RLN palsy**												
11	observational studies	not serious	not serious	not serious	not serious	none	39/855 (4.6%)	41/1316 (3.1%)	**OR 1.33** (0.83 to 2.15)	**10 more per 1,000** (from 5 fewer to 34 more)	⊕⊕◯◯ Low	
**Permanent RLN palsy**												
12	observational studies	not serious	not serious	not serious	not serious	none	4/885 (0.5%)	6/1339 (0.4%)	**OR 1.74** (0.26 to 11.62)	**3 more per 1,000** (from 3 fewer to 45 more)	⊕⊕◯◯ Low	
**Transient hypocalcemia**												
8	observational studies	not serious	not serious	not serious	not serious	none	60/737 (8.1%)	69/888 (7.8%)	**OR 0.81** (0.56 to 1.17)	**14 fewer per 1,000** (from 33 fewer to 12 more)	⊕⊕◯◯ Low	
**Permanent hypocalcemia**												
8	observational studies	not serious	not serious	not serious	not serious	strong association	2/737 (0.3%)	11/888 (1.2%)	**OR 0.31** (0.04 to 2.64)	**9 fewer per 1,000** (from 12 fewer to 20 more)	⊕⊕⊕◯ Moderate	
**Hospital stay**												
6	observational studies	not serious	not serious	not serious	not serious	none	439	402	-	MD **0.27 higher** (0.14 higher to 0.39 higher)	⊕⊕◯◯ Low	
**Pain VAS**												
4	observational studies	not serious	not serious	serious[a]	not serious	none	387	374	-	MD **1.41 lower** (2.79 lower to 0.03 lower)	⊕◯◯◯ Very low	

CI: confidence interval; **MD:** mean difference; **OR:** odds ratio

Explanations

a. subjective assessment
