# Supplementary material for: Transoral endoscopic thyroidectomy vestibular approach as a safe and feasible alternative to open thyroidectomy: a systematic review and meta-analysis
Source: Int J Surg. 2023 May 10;109(8):2467–77. doi: 10.1097/JS9.0000000000000444 (PMC10442077; doi:10.1097/JS9.0000000000000444)
Supplement: Supplementary file 5 [file js9-109-2467-s005.pdf]

Summary of findings:

TOETVA compared to OT for thyroidectomy

Patient or population: thyroidectomy
Setting:
Intervention: TOETVA
Comparison: OT

Outcomes	Anticipated absolute effects* (95% CI)		Relative effect (95% CI)	№ of participants (studies)	Certainty of the evidence (GRADE)	Comments
	Risk with OT	Risk with TOETVA				
Operation time		MD **55.19 higher** (39.15 higher to 71.23 higher)	-	2231 (12 observational studies)	⊕⊕◯◯ Low	
Transient RLN palsy	31 per 1,000	**41 per 1,000** (26 to 65)	**OR 1.33** (0.83 to 2.15)	2171 (11 observational studies)	⊕⊕◯◯ Low	
Permanent RLN palsy	4 per 1,000	**8 per 1,000** (1 to 50)	**OR 1.74** (0.26 to 11.62)	2224 (12 observational studies)	⊕⊕◯◯ Low	
Transient hypocalcemia	78 per 1,000	**64 per 1,000** (45 to 90)	**OR 0.81** (0.56 to 1.17)	1625 (8 observational studies)	⊕⊕◯◯ Low	
Permanent hypocalcemia	12 per 1,000	**4 per 1,000** (1 to 32)	**OR 0.31** (0.04 to 2.64)	1625 (8 observational studies)	⊕⊕⊕◯ Moderate	
Hospital stay		MD **0.27 higher** (0.14 higher to 0.39 higher)	-	841 (6 observational studies)	⊕⊕◯◯ Low	
Pain VAS		MD **1.41 lower** (2.79 lower to 0.03 lower)	-	761 (4 observational studies)	⊕◯◯◯ Very low[a]	

***The risk in the intervention group** (and its 95% confidence interval) is based on the assumed risk in the comparison group and the **relative effect** of the intervention (and its 95% CI).

CI: confidence interval; **MD:** mean difference; **OR:** odds ratio

GRADE Working Group grades of evidence
High certainty: we are very confident that the true effect lies close to that of the estimate of the effect.
Moderate certainty: we are moderately confident in the effect estimate: the true effect is likely to be close to the estimate of the effect, but there is a possibility that it is substantially different.
Low certainty: our confidence in the effect estimate is limited: the true effect may be substantially different from the estimate of the effect.
Very low certainty: we have very little confidence in the effect estimate: the true effect is likely to be substantially different from the estimate of effect.

Explanations

a. subjective assessment
